# Dr. Henry Herrick

**Published:** 1901-03

**Authors:** 


Dr. Henry J. Herrick, a widely known physician and surgeon, of Cleveland, Ohio, died January 28, 1901. He was for many years professor of the medical department of the Western Reserve University. He was a companion of the Military Order of the Loyal Legion, and served as surgeon-general of the Ohio National Guard during Governor Foraker's administration.

